# Effects of load and maintenance duration on the time course of information encoding and retrieval in working memory: from perceptual analysis to post-categorization processes

**DOI:** 10.3389/fnhum.2014.00165

**Published:** 2014-04-01

**Authors:** Diego Pinal, Montserrat Zurrón, Fernando Díaz

**Affiliations:** Department of Clinical Psychology and Psychobiology, Universidade de Santiago de CompostelaSantiago de Compostela, Spain

**Keywords:** event-related potentials, working memory, delayed match-to-sample, encoding, retrieval, memory load, maintenance duration, eLORETA

## Abstract

Working memory (WM) involves three cognitive events: information encoding, maintenance, and retrieval; these are supported by brain activity in a network of frontal, parietal and temporal regions. Manipulation of WM load and duration of the maintenance period can modulate this activity. Although such modulations have been widely studied using the event-related potentials (ERP) technique, a precise description of the time course of brain activity during encoding and retrieval is still required. Here, we used this technique and principal component analysis to assess the time course of brain activity during encoding and retrieval in a delayed match to sample task. We also investigated the effects of memory load and duration of the maintenance period on ERP activity. Brain activity was similar during information encoding and retrieval and comprised six temporal factors, which closely matched the latency and scalp distribution of some ERP components: P1, N1, P2, N2, P300, and a slow wave. Changes in memory load modulated task performance and yielded variations in frontal lobe activation. Moreover, the P300 amplitude was smaller in the high than in the low load condition during encoding and retrieval. Conversely, the slow wave amplitude was higher in the high than in the low load condition during encoding, and the same was true for the N2 amplitude during retrieval. Thus, during encoding, memory load appears to modulate the processing resources for context updating and post-categorization processes, and during retrieval it modulates resources for stimulus classification and context updating. Besides, despite the lack of differences in task performance related to duration of the maintenance period, larger N2 amplitude and stronger activation of the left temporal lobe after long than after short maintenance periods were found during information retrieval. Thus, results regarding the duration of maintenance period were complex, and future work is required to test the time-based decay theory predictions.

## INTRODUCTION

Working memory (WM) is defined as the capacity to hold in mind for brief periods of time small amounts of information that are no longer available in the environment (Baddeley, 2012). This capacity is supported by synaptic changes, neural firing and synchronous activity in a network of brain regions (Buzsáki and Draguhn, 2004), mainly involving frontal, parietal, and temporal lobes (Klingberg, 2006; Linden, 2007).

A model linking cerebral activity and the cognitive events involved in WM has recently been proposed (Jonides et al., 2008). This model considers three cognitive events: information encoding in memory, information maintenance, and information retrieval. These cognitive events are, in turn, composed of different subprocesses that vary depending on the nature of the task. The above-mentioned authors also discuss two aspects that modulate and limit WM, namely, memory load and the mechanisms that cause forgetting. Memory load, which is defined as the amount of information that participants must hold in mind, is determined by both the amount and the complexity of the stimuli (see Luck and Vogel, 1997; Alvarez and Cavanagh, 2004). Jonides and colleagues also discuss a time-based decay theory that was proposed to explain the mechanisms that lead to forgetting: in this theory, the mere passage of time is considered to produce the enfeeblement of memory traces and thus the disruption or complete loss of the memory.

In order to exemplify their model, Jonides et al. (2008) used a delayed matching to sample (DMS) task. This type of task enables the isolated study of the different cognitive events involved in the model, i.e., encoding, maintenance and retrieval. Trials consisted of presentation of a sample stimulus to be memorized (encoding), a variable period of time in which information about the sample stimulus must be held in mind (maintenance) and presentation of a probe stimulus that subjects must compare (e.g., same/different, absent/present, …) with the sample stimulus (retrieval and comparison) (John et al., 1996; van der Ham et al., 2010). Moreover, in DMS tasks, the memory load can be manipulated by varying the number or complexity of the stimuli. The duration of the maintenance period can also be varied, thus manipulating the weakening of memory traces due to time-based decay.

However, because the model is based on the results of functional neuroimaging studies, which have excellent spatial resolution but rather low temporal resolution, it lacks detailed specification of the time course of brain activity during encoding, maintenance and retrieval. The event-related potentials (ERP) technique is a potentially useful tool for studying the time course of brain activity as the temporal resolution is in the order of milliseconds. The technique should also be particularly suitable for studying encoding and retrieval events, as it has been used to assess brain activity that displays stable time relationships to a definable reference event, such as stimulus presentation. Furthermore, several ERP components have been shown to be sensitive to manipulations of memory load and of the duration of maintenance period (Houlihan et al., 1998; Kok, 2001; Morgan et al., 2008; van der Ham et al., 2010; Soria Bauser et al., 2011).

Regarding the effects of the duration of maintenance period on ERP components, van der Ham et al. (2010) reported that during information retrieval in a DMS task (upon presentation of the probe stimulus), the amplitude of the N2 component is larger in frontal and parietal sites after a long maintenance period than after a short maintenance period (i.e., 5000 and 500 ms or 2000 ms, respectively). The authors related the larger N2 after long maintenance periods to greater difficulty in stimulus discrimination and greater WM demand exerted by long maintenance period conditions (LMPs; see also, Barch et al., 1997). However, it is not clear whether changes in the duration of the maintenance period affect other components of the ERP wave (besides N2) and, therefore, other subprocesses pertaining to the time course of WM retrieval (besides stimulus discrimination).

Several ERP studies have analyzed the effects of memory load variations. In this context, memory load has been shown to modulate the amplitude of ERP components related to stimulus perceptual processing, such as P1 and N1 (Taylor, 2002; Morgan et al., 2008), as well as the amplitude and latency of ERP components associated with stimulus discrimination and classification and with decision making, such as N2 and P300 (García-Larrea and Cézanne-Bert, 1998; Kok, 2001; Polich, 2007; Morgan et al., 2008).

Nevertheless, there is some controversy about how memory load modulates the amplitude of some ERP components during WM encoding and retrieval. For instance, some authors have reported that the P300 amplitude was larger in high load (HL) conditions than in low load (LL) conditions during information encoding (upon presentation of a sample stimulus in a DMS task) (Houlihan et al., 1998; Studer et al., 2010), while other authors have found the opposite (Morgan et al., 2008; Soria Bauser et al., 2011). Regarding information retrieval (upon presentation of the probe stimulus in a DMS task), several authors have reported lower P300 amplitude with high memory load than with low memory load (Houlihan et al., 1998; Morgan et al., 2008); however, other authors failed to find such modulation in P300 amplitude in relation to memory load (e.g., Studer et al., 2010).

In the present study, we recorded EEGs while participants completed a DMS task. First, in order to determine the time course structure of encoding and retrieval in WM, we used a temporal principal component analysis (PCA) to decompose the ERP waveforms produced in response to sample and probe stimuli into their latent temporal factors (TF), which identify the ERP components. Second, we created two memory load conditions in order to study the effect of this variable on amplitude and peak latency of ERP components during WM encoding and retrieval. Similarly, we established two maintenance period durations in order to establish the effect of maintenance duration on amplitude and peak latency of ERP components during WM retrieval. Third, we used low resolution brain electromagnetic tompography (LORETA) to examine the effects of manipulation of memory load and duration of the maintenance period on the time course of fronto-parietal network activation.

On the basis of the findings of previous ERP studies, it is expected that the time course structure of both cognitive events (encoding and retrieval) might involve ERP components associated with perceptual processing, such as P1 and N1, as well as components related to feature extraction and stimulus discrimination and categorization, such as P2, N2, and P300. It probably also includes processes that occur after stimulus categorization. Regarding the experimental manipulations, higher levels of memory load and longer durations of the maintenance period will probably produce a decrease in task performance. Furthermore, we expect modulation of P300 amplitude and latency to occur with higher memory loads. We also predict an increase in N2 amplitude with longer durations of the maintenance period.

## MATERIALS AND METHODS

### SAMPLE

Twenty nine healthy volunteers (20 female; mean age: 20.5 ± 2.84 years) were recruited from students at the University of Santiago de Compostela (USC). All participants except three were right handed, as assessed by the Edinburgh Handedness Inventory (Oldfield, 1971). All had normal or corrected to normal vision. Participants were asked to abstain from consuming drinks that act as stimulants or depressants for several hours before the session. The study protocols were approved by the USC Ethics Committee, and all participants gave their informed consent prior to the experimental session.

### TASK AND STIMULI

**FIGURE 1:**
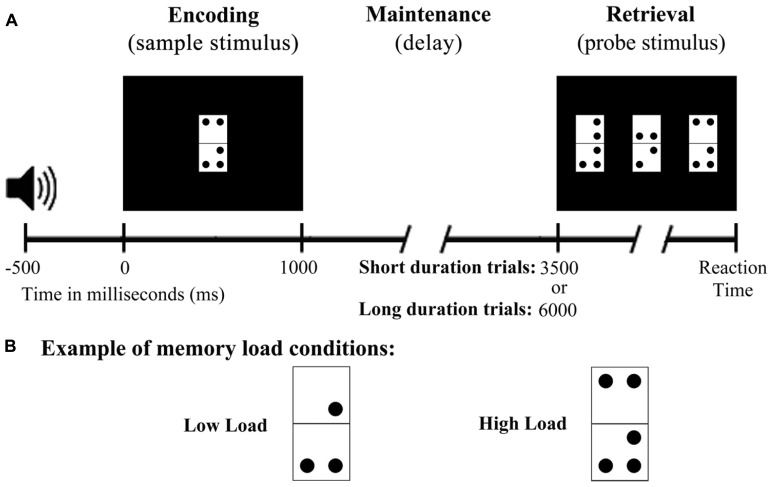
**Diagram of the delayed match to sample task used in the study. (A)** Schematic illustration of the time course of a single trial. **(B)** Example stimulus from both memory load conditions.

**Figure 1A** shows an example of the DMS task trials. Each trial began with a 50 ms warning tone (1000 Hz), and 500 ms later the sample stimulus was presented on screen for 1000 ms. This was followed by a stimulation-free period lasting 2500 or 5000 ms, and the probe stimuli were then presented on screen until execution of the participant’s response or, if there was no response, for a maximum duration of 3000 ms. The inter-trial interval (between the participant’s response and the warning tone) was 800 ms. To reduce ocular artifacts, a fixation cross was presented in the center of the screen whenever there was no stimulation. Stimulus presentation and response recording were controlled by Presentation® software (Neurobehavioral Systems, Inc., Albany, CA, USA).

All stimuli were presented against a black background on a 19” computer monitor (100 Hz refresh rate) placed at a distance of 1 m from the participant’s eyes. The sample stimulus consisted of an adapted rectangular domino tile (8 cm long × 4 cm wide or 4.58° × 2.29° of visual angle) composed by two vertically arranged white squares of equal size (**Figure 1B**). Between 0 and 3 dots were positioned in each half of the tile in one of four different locations, one per corner. The dots were black and were located 0.5 cm from the edges of the tile and 1 cm from each other.

The probe stimulus comprised three tiles of the same structure as those used in the sample stimulus. The tiles were presented in the center and on the right and left of the computer screen at a distance of 4 cm from each other. Only one of the tiles (target) in the probe stimulus was identical to that presented as the sample stimulus, and its position was counterbalanced so that it did not appear in the same position in more than three consecutive trials. The use of three domino tiles in the probe stimulus was intended to ensure active retrieval of the sample stimulus information and to avoid a response based solely on a feeling of familiarity.

Participants were asked to memorize (encode) the configuration of dots in the domino used as sample stimulus, to hold it in mind during the maintenance period, and to retrieve it in order to identify the target among the three domino tiles that formed the probe stimulus. Participants were also instructed to press, as quickly and accurately as possible, the button corresponding to the position of the target in the screen (left, center, or right) using a respond pad (Cedrus®, model RB-530) with three horizontally arranged buttons. Participants were instructed to use their left hand to press the left button and the right hand to press the other two buttons. They were also trained in the task prior to the EEG recording to confirm that they had understood the instructions. The complete DMS task comprised 200 trials divided into two blocks, and the execution time was no longer than 30 min, including a 5 min break between blocks.

The two task blocks differed in the memory load of their stimuli. The first block was always the LL block, which consisted of 90 trials with dominos with 2 or 3 dots in total (76 possible combinations). The second block was always the HL block, which consisted of 110 trials with dominos with 4 or 5 dots in total (116 possible combinations). In this block, the use of domino tiles with two dots in each half was limited to those with the dots in one half forming a diagonal in order to avoid simple and easily verbalized configurations (100 possible combinations). As the number of possible combinations was less than the number of trials in each block, 20% of the trials in each block included as sample stimulus a domino that was also present in another trial. In addition, more trials were included in the HL block than in the LL block to ensure a good signal to noise ratio in the ERPs, as it was expected that participants would make more errors in the HL condition than in the LL condition.

Finally, half of the trials in each block constituted the short maintenance period condition (SMP; maintenance period of 2500 ms). The other half of trials in each block formed the LMP condition (maintenance period of 5000 ms). The probability of appearance of both duration times was the same (50%) and both were distributed pseudo-randomly, to avoid more than five consecutive trials having the same maintenance period duration.

### EEG RECORDING

During the experimental session, the participants sat in a comfortable armchair inside a Faraday chamber with attenuated levels of noise and light. The EEG signal was recorded at 49 active electrodes inserted in a cap (Easycap GmbH) and placed according to the 10–10 international system. The EOG activity was monitored by placing two electrodes in the outer canthi of both eyes (HEOG) and another two electrodes placed infra and supraorbitally to the right eye (VEOG). Impedances were maintained below 10 kΩ. Fronto-polar ground was used and all electrodes were referenced to an electrode in the nose tip. The EEG signal was analogically filtered between 0.01 and 100 Hz, sampled at 500 Hz and digitally recorded for off-line analyses.

Recorded data were passed through a digital phase-shift free Butterworth filter with the high cut-off frequency at half power (-3 dB) set in 30 Hz (12 dB/octave roll-off). A notch-filter centered at 50 Hz was also applied to avoid any contamination of electrical line noise. Ocular and muscular artifacts were corrected by independent component analysis (ICA), using the Infomax algorithm, as implemented in Brain Vision Analyzer (v.2 Brain Products GmbH). Semi-automatic artifact rejection was also applied. The EEG data were then segmented into epochs from 200 ms prior to stimulus to 1000 ms post-stimulus, and the pre-stimulus period was defined as baseline. Only epochs corresponding to correctly answered trials entered further analyses.

Six averaged ERP waves were obtained for each subject: two corresponding to the sample stimulus related activity (one for LL condition and other for HL condition) and another four corresponding to the probe stimulus related activity (one for the combination of LL and SMP conditions, one for the combination of LL and LMP, one for the combination of HL and SMP, and another for the combination of HL and LMP).

### BEHAVIORAL AND ELECTROPHYSIOLOGICAL DATA

Proportion of correct responses and reaction times (RT; for correct responses only) were registered as behavioral data for each one of the four experimental conditions (LL-SMP, LL-LMP, HL-SMP, and HL-LMP). Both measures were combined in the inverse efficiency (IE) score, which is equal to the mean RT divided by the proportion of correct responses (Townsend and Ashby, 1978). Accordingly, increases in RT or decreases in the proportion of correct responses would result in increases in the IE score; hence, higher IE scores reflect poorer performance than lower scores. Furthermore, this measure can be considered as a “corrected reaction time,” which avoids possible speed-accuracy tradeoffs or criterion shifts (Kennett et al., 2001; Jacques and Rossion, 2007; Collignon et al., 2008). The IE was calculated for each subject and each condition.

Temporal principal components analysis (tPCA) was applied to sample stimulus ERP data for LL and HL conditions and to probe stimulus ERP data for the LL-SMP, LL-LMP, HL-SMP, and HL-LMP conditions. tPCA is a factor-analytic procedure that uses eigenvalue decomposition to extract linear combinations of variables (latent factors) in order to account for patterns of covariance in a parsimonious fashion (Dien and Frishkoff, 2005). In ERP analysis, the main source of covariance is assumed to rely on the ERP components, defined as characteristic features of the waveform that are spread across multiple time points and electrodes (Donchin and Coles, 1991). In other words, digitalized points that comprise an ERP component are believed to increase or decrease together, so that their dynamics correlate or covary. Therefore, tPCA retained and rotated factors are considered to reflect pure signals (i.e., brain activity), which would ideally present a one-to-one relation with the latent ERP components. Consequently, tPCA was used to detect features of the ERP waveform that might escape visual inspection due to overlapping and summation of ERP components (Dien, 1998; Dien and Frishkoff, 2005; Dien et al., 2005).

In the present study, the covariance matrix was used in both tPCAs. The number of factors retained from the unrotated factors solution was based on the scree test (Catell, 1966). Furthermore, retained factors were submitted to Promax rotation, which improves the accuracy of the results and avoids problems such as the misallocation of variance (Dien, 1998). tPCA analysis yields two matrices: one comprising the factor loadings, which provides information about the temporal characteristics of the factors, and another comprising the factor scores, which provides information about the magnitude of each factor for each electrode and condition. Therefore, the values of these matrices can be used to establish the relationship between tPCA factors and ERP components. Moreover, as factor scores are transformations of the original voltage values (Dien, 1998), they were used as alternative measures of the amplitude of the ERP components for each condition, electrode and participant.

**FIGURE 2:**
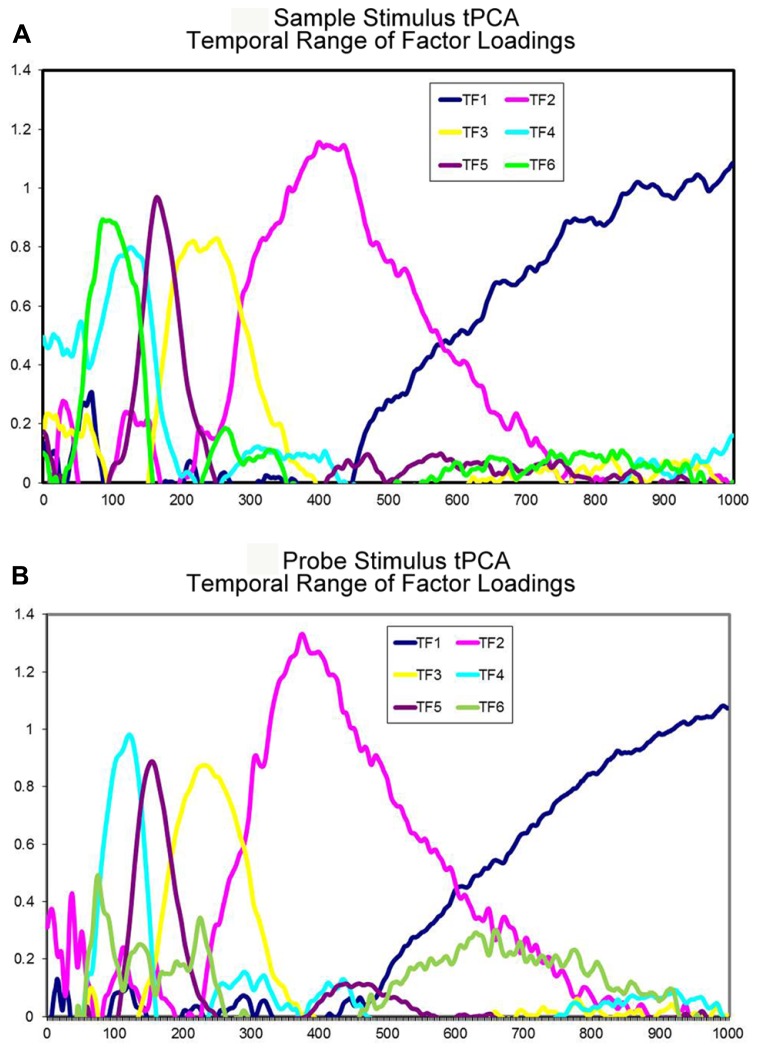
**Temporal principal components analysis extracted factors. (A)** Time course of the different temporal factors extracted in the tPCA with sample stimulus related data. **(B)** Time course of the different temporal factors extracted in the tPCA with probe stimulus related data.

The tPCA applied to sample stimulus ERP data identified six TF accounting for 92.87% of the variance (**Figure 2A**). Considering the temporal range of the factor loadings and the distribution of the factor scores among the electrodes, the TFs were associated with the following ERP components: TF6 corresponded to P1 component, as the largest loadings were observed between 75 and 125 ms post-stimulus and the largest scores at occipital electrodes (Oz, O2, and O1). TF4 was associated with the N1 component, as the maximum loadings were observed between 95 and 150 ms post-stimulus and the maximum negative scores were obtained at central and parietal sensors (Cz, CPz, and Pz). TF5 was related to the P2 component as the highest loadings occurred between 150 and 195 ms post-stimulus and the highest scores were obtained at occipital and central electrodes (Oz, O2, and Cz). TF3 was associated with the N2 component as the highest loadings extended from 195 to 275 ms post-stimulus and the largest negative scores were located at parietal sites (P9, P10, and TP9). TF2 corresponded to the P300 component, as its maximum loadings were obtained between 310 and 475 ms post-stimulus and the maximum scores were obtained at parieto-occipital electrodes (P3, Pz, and Oz). Finally, TF1 was associated with a positive slow wave (PSW) because the factor loadings were maximal between 550 ms and the end of the recording epoch, whereas the maximal scores were located at central sites (C3, C1, and Cz).

Regarding the tPCA applied to probe stimulus ERP data, six TF explaining 95.44% of the variance were identified (**Figure 2B**). On the basis of the temporal range of factor loadings and on the distribution of factor scores across scalp electrodes, the TFs were associated with the following ERP components: TF6 was associated with the P1 component as the largest loadings were observed between 65 and 100 ms post-stimulus and the largest scores were obtained at parietal and occipital electrodes (Pz, Oz, P4). TF4 was associated with the N1 component, as the maximum loadings occurred between 85 and 150 ms post-stimulus and the most negative scores were obtained at central electrodes (Cz, CPz, and C1). TF5 was associated with the P2 component, as the highest loadings extended from 135 to 190 ms post-stimulus and the highest scores were obtained at fronto-central sites (Cz, FCz, and Fz). TF3 was associated with the N2 component, as the maximal loadings were observed between 190 and 285 ms post-stimulus and the maximal negative scores were observed at parieto-occipital sites (P9, PO7, and P10). TF2 corresponded to the P300 component, as the largest loadings extended from 320 to 475 ms and the largest scores were recorded at occipito-parietal electrodes (Pz, Oz, and O2). Finally, TF1 was associated with a negative slow wave (NSW) because the maximal loadings were observed between 600 ms and the end of the analyzed epoch, while the maximal negative scores were recorded at fronto-central electrodes (FCz, C1, and Fz).

For the statistical analyses of sample stimulus and probe stimulus ERP data, amplitude and latency values of the ERP components were obtained. The amplitude was calculated as the mean factor scores from the three electrodes showing the maximum values for TF1 (slow waves), TF2 (P300), TF3 (N2), TF4 (N1), TF5 (P2), and TF6 (P1). In addition, we measured the peak latencies of P1, N1, P2, N2, and P300 on ERP waveforms at the same three electrodes as the factor scores. The mean values recorded for each component across these three electrodes were used in the statistical analyses.

In addition, exact low-resolution brain electromagnetic tomography (eLORETA) software was used to identify the cortical origin of potential differences in electrical brain activity between both memory load conditions (LL and HL) in sample stimulus EEG data, and between LL and HL, as well as between both maintenance period duration conditions (SMP and LMP) in EEG data for the probe stimulus. In all three comparisons, the first 1000 ms post-stimulus presentations were used in the calculations.

Exact low-resolution brain electromagnetic tomography uses the scalp recorded voltage data of all active electrodes for each condition and participant to estimate the neural origin of potential differences between two experimental conditions. This software is a discrete, 3D distributed, linear, weighted minimum norm inverse solution. The particular weights used in this software endow the tomography with the property of exactly localizing test point sources, yielding images of current density with exact localization, albeit with low spatial resolution (i.e., neighboring neuronal sources will be highly correlated). Therefore, it calculates inverse solutions by identifying the smoothest of all possible 3D current density distributions that would explain the surface potentials (Pascual-Marqui et al., 1994; Pascual-Marqui, 1999). The eLORETA computations were made in a realistic head model (Fuchs et al., 2002) based on the Montreal Neurological Institute (MNI152) template (Mazziotta et al., 2001). Furthermore, the three-dimensional solution space was restricted to cortical gray matter, while the intracerebral volume was partitioned in 6239 voxels at 5 mm spatial resolution. Thus, eLORETA generates functional images that represent in the neuroanatomical MNI space, the brain regions that showed activation differences between two experimental conditions.

### STATISTICAL ANALYSES

To assess the possible effects of memory load and maintenance period duration on the behavioral performance in the DMS task, the IE scores were subjected to a repeated measure analysis of variance (ANOVA) with two within-subjects factors: Memory Load (LL and HL) and Maintenance Period Duration (SMP and LMP).

Regarding brain activity, the effects of memory load during WM encoding (during sample stimulus presentation) were evaluated by Student’s *t*-tests for paired samples. These tests compared TF scores (as an alternative measure of amplitude) and peak latencies of each ERP component between the two memory load conditions (LL and HL). Factor scores and peak latencies were averaged across three electrodes before being entered in the analysis: Oz, O2, and O1 electrodes for TF6-P1; Cz, CPz, and Pz electrodes for TF4-N1; Oz, O2, and Cz electrodes for TF5-P2; P9, P10 and TP9 electrodes for TF3-N2; P3, Pz, and Oz electrodes for TF2-P300; and C3, C1, and Cz electrodes for TF1 (PSW; only factor scores).

The effects of memory load and the duration of the maintenance period on brain activity during information retrieval (during presentation of the probe stimulus) were assessed by repeated measures ANOVAs with the within-subjects factors: Memory Load (LL and HL) and Maintenance Period Duration (SMP and LMP). The dependent variables in the analyses were the factor scores (as an alternative measure of amplitude) and the peak latencies of the ERP components corresponding to the four experimental conditions created by the combination of the two levels of both factors. Data were averaged across three electrodes before being entered in the analysis: Pz, Oz, and P4 electrodes for TF6-P1; Cz, CPz, and C1 electrodes for TF4-N1; Cz, FCz, and Fz electrodes for TF5-P2; P9, PO7, and P10 electrodes for TF3-N2; Pz, Oz, and O2 electrodes for TF2-P300; and FCz, C1 and Fz electrodes for TF1 (NSW; only factor scores).

In all the repeated measures ANOVAs carried out in the present study, Greenhouse-Geisser correction was applied whenever there was violation of the sphericity assumption, and the Bonferroni test was applied in *post hoc* comparisons whenever necessary. All effects were considered significant at *p* ≤ 0.05.

Additionally, scalp voltage data recorded at all active electrodes for both memory load conditions during encoding, and for both memory load as well as both maintenance period duration conditions during retrieval were subjected to a map-to-map comparison of electrical activity (TANOVA) via the maps dissimilarity measure (Lehmann et al., 1987) as implemented by eLORETA software. This measure allows estimation of the temporal points when brain electrical activity differed between two experimental conditions. Therefore, the TANOVA results are temporal intervals that enclose significant differences in the topography of brain activity between two experimental conditions. The statistical significance (*p* ≤ 0.05) for each map pair was evaluated non-parametrically by use of a randomization test that also corrects for multiple comparisons (for procedures, see Strik et al., 1998). Thus, in order to assess the effects of memory load and duration of maintenance period, three TANOVAs were applied: the first compared the scalp recorded voltage data of all active electrodes between both memory load conditions (LL and HL) during sample stimulus processing (information encoding), the second compared the scalp recorded voltage data of LL and HL conditions during probe stimulus processing (information retrieval), and the last compared the voltage data recorded in all scalp active electrodes between both maintenance period duration conditions (SMP and LMP) during probe stimulus processing (information retrieval).

Then, voltage data pertaining to the time windows that reach significance in the TANOVAs that compare both memory load conditions during encoding, and both memory load as well as both maintenance period duration conditions during retrieval was subjected to non-parametric analysis of functional images as implemented in eLORETA. To complete these analyses, eLORETA estimates the smoothest 3D current density distributions that would explain the surface potentials, and it uses the paired-samples log of ratio of averages statistic (similar to the log of *F*-ratio statistic) to compare the estimation made for two experimental conditions. In these analyses, the statistical significance is tested with a randomization test; hence, the statistical non-parametric mapping (SNPM) methodology used corrects for multiple comparisons and does not require any assumption of Gaussianity (Nichols and Holmes, 2002). Consequently, SNPM was used to localize the brain regions showing significant activation differences in the temporal intervals indicated as significant in the TANOVAs.

## RESULTS

### BEHAVIORAL RESULTS

Inverse efficiency scores analysis revealed a significant main effect for Memory Load [*F*(1,28) = 39.74, *p* < 0.001] and a significant interaction between Memory Load and Maintenance Period Duration [*F*(1,28) = 5.95, *p* ≤ 0.021; **Table 1**]. *Post hoc* comparisons revealed that IE scores were significantly larger in the HL than in the LL condition for both maintenance period durations (*p* < 0.001).

**Table 1:** Summary of behavioral measurements.

	LL			HL		
	IE	RT	Correct responses	IE	RT	Correct responses
SMP	1156.6 (180.1)	1060.5 (160.5)	91.8 (4.4)	1339.8 (234.4)	1214.6 (182.8)	91.1 (5.1)
LMP	1173.9 (203.6)	1111.2 (185.3)	94.9 (4.7)	1300.6 (266.1)	1182.1 (208.8)	91.3 (4.4)

*Mean inverse efficiency (IE) scores and reaction times (RT; both in ms), and percentage of correct responses with their standard deviations (in brackets). LL, low memory load condition; HL, high memory load condition; SMP, short maintenance period condition, LMP, long maintenance period condition.*

### ELECTROPHYSIOLOGICAL RESULTS

#### Effects of memory load on electrical brain activity during information encoding (sample stimulus)

**FIGURE 3:**
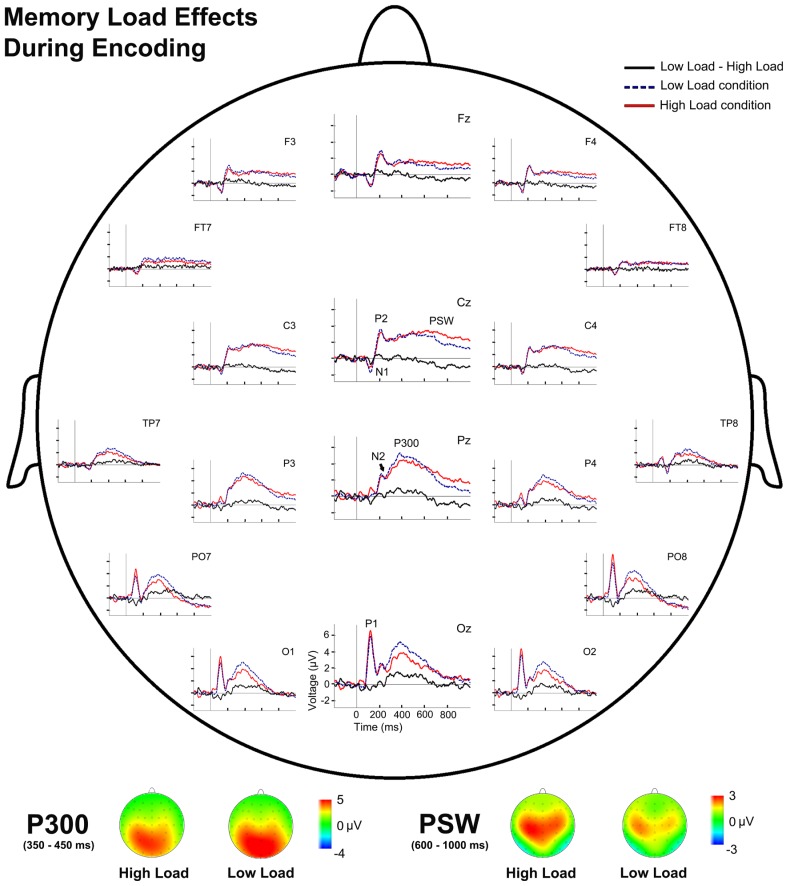
**Event-related potentials waveforms during sample stimulus processing and voltage maps for components modulated by WM load.** (Top) Topographic plot of the ERP waveforms for both WM load conditions and their difference (LL-HL) during encoding at several scalp electrodes. (Bottom) P300 and PSW voltage maps for both WM load conditions during encoding.

Analysis of the effects of memory load during WM encoding revealed significantly lower TF2 factor scores in the HL condition than in the LL condition [*t*(28) = 3.79, *p* = 0.001], which corresponded to a lower P300 amplitude in the HL condition than in the LL condition. By contrast, factor scores were significantly higher in the HL than in the LL condition for TF1 [*t*(28) = -2.04, *p* = 0.051], and therefore the amplitude of the PSW component was larger in the HL than in the LL condition (**Figure 3**).

**FIGURE 4:**
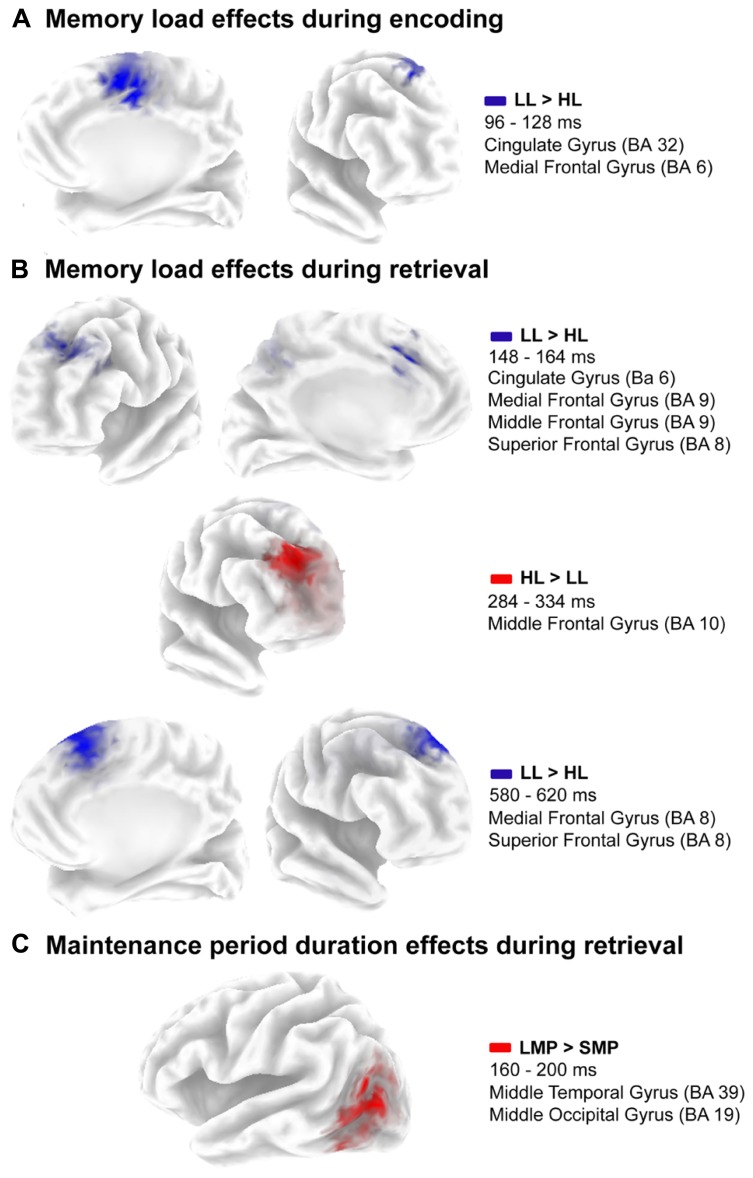
**Source location of estimated activation differences. (A)** Cluster of voxels where the maximal difference between low load condition and high load condition is located during codification. **(B)** Cluster of voxels where the maximal difference between low load and high load condition is located during retrieval. **(C)** Cluster of voxels where the maximal difference between short maintenance period duration and long maintenance period duration is located during retrieval.

Finally, brain activity differed significantly between both memory load conditions from 96 to 128 ms post-stimulus (*p* = 0.051). SNPM analysis for that time interval showed significantly higher activation in the anterior cingulate (BA 32) and medial frontal (BA 6) gyri of the right hemisphere for the LL condition than for the HL condition (**Figure 4A**). Coordinates of the voxels showing maximal difference, *t* values and the associated p values are shown in **Table 2**.

**Table 2:** Voxels showing maximal differences in activation between conditions.

Coordinates			Brain region	Statistics	
*X*	*Y*	*Z*		*t* value	Assoc. *p*
**Memory load during encoding**
5	15	45	Cingulate gyrus (BA 32)	0.689	<0.05
5	15	50	Medial frontal gyrus (BA 6)	0.682	<0.05
**Memory load during retrieval (148–164 ms)**
-15	25	35	Medial frontal gyrus (BA 9)	1.025	<0.01
-15	25	40	Cingulate gyrus (Ba 6)	0.995	<0.01
-30	20	35	Middle frontal gyrus (BA 9)	0.954	<0.01
-20	40	50	Superior frontal gyrus (BA 8)	0.932	<0.01
**Memory load during retrieval (284–334 ms)**
30	45	25	Middle frontal gyrus (BA 10)	1.090	<0.05
**Memory load during retrieval (580–620 ms)**
15	45	45	Superior frontal gyrus (BA 8)	0.870	<0.01
5	40	45	Medial frontal gyrus (BA 8)	0.856	<0.01
**Maintenance period duration during retrieval**
-50	-75	20	Middle temporal gyrus (BA 39)	2.079	<0.01
-50	-77	13	Middle occipital gyrus (BA 19)	2.029	<0.05

*MNI coordinates of the voxels showing the maximal difference between conditions, the correspondent brain region and Brodmann area, the *t* value and the associated *p*.*

#### Effects of memory load on electrical brain activity during information retrieval (probe stimulus)

**FIGURE 5:**
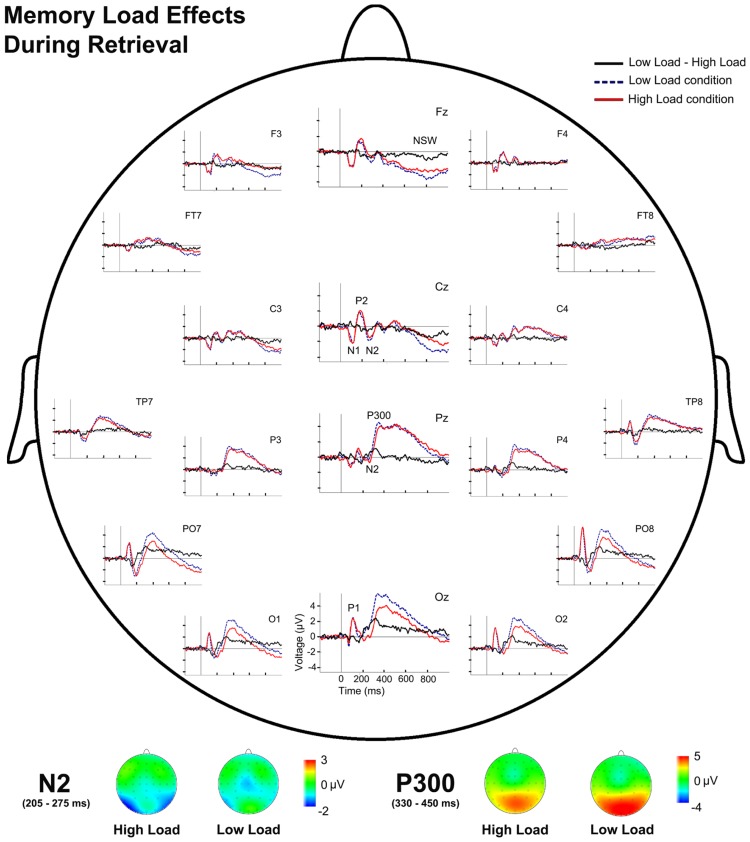
**Event-related potentials waveforms during probe stimulus processing and voltage maps for components modulated by WM load.** (Top) Topographic plot of the ERP waveforms for both WM load conditions and their difference (LL-HL) during retrieval at several scalp electrodes. (Bottom) N2 and P300 voltage maps for both WM load conditions during retrieval.

Regarding the effects of memory load during information retrieval, repeated measures ANOVAs revealed a main effect of Memory Load for TF3 [*F*(1,28) = 38.66, *p* = 0.001] and for TF2 [*F*(1,28) = 10.72, *p* = 0.003]. *Post hoc* comparisons showed significantly more negative factor scores in the HL condition than in the LL condition for TF3 (N2; *p* < 0.001) and also significantly lower factor scores for the HL than for the LL for TF2 (P300; *p* = 0.003). Therefore, the ANOVA revealed higher amplitude of N2 in the HL condition than in the LL condition, but smaller P300 amplitude in HL than in LL (**Figure 5**).

Regarding the latencies of the ERP components, the repeated measures ANOVA revealed a significant main effect of Memory Load [*F*(1,28) = 7.71, *p* = 0.01] and a significant effect of the interaction between Memory Load and Maintenance Period Duration [*F*(1,28) = 5.53, *p* = 0.026] on P300 latency. Paired comparisons revealed a significantly longer P300 latency for the HL than the LL condition for short maintenance periods (*p* = 0.001).

Furthermore, brain activity differed significantly between memory load conditions in several time windows (*p* = 0.001). Greater activation in the LL condition than in the HL condition was observed in the time intervals between 148 and 164 ms and between 580 and 620 ms post-stimulus (**Figure 4B**). The differences in activation were identified in the left superior (BA 8), medial (BA 8, 32, and 6) and middle (BA 9 and 8) frontal gyri and in the left anterior cingulate (BA 32), in the 148 to 164 ms post-stimulus interval; and in the right superior (BA 8) and medial (BA 6) frontal gyri, in the 580 to 620 ms post-stimulus interval. Conversely, the activation was significantly lower in the right middle frontal gyrus (BA 10) between 284 and 334 ms post-stimulus for the LL condition than for the HL condition. Coordinates of the voxels showing maximal differences as well as *t* values and associated *p* values are provided in **Table 2**.

#### Effects of maintenance period duration on electrical brain activity during information retrieval (probe stimulus)

**FIGURE 6:**
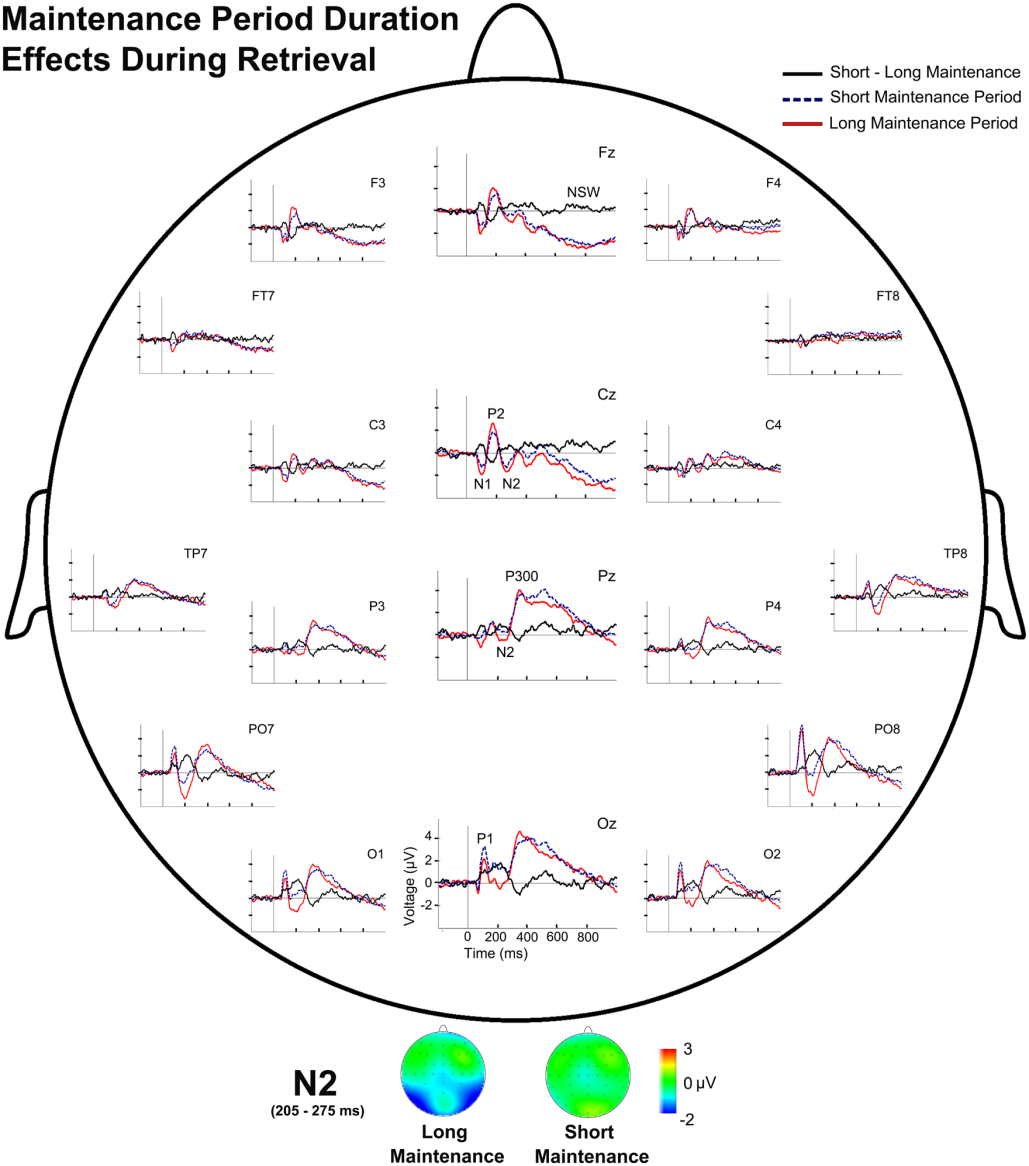
**Event-related potentials waveforms during probe stimulus processing and voltage maps for components modulated by maintenance period duration.** (Top) Topographic plot of the ERP waveforms for both maintenance period duration conditions and their difference (SMP-LMP) during retrieval at several scalp electrodes. (Bottom) N2 voltage map for both maintenance period duration conditions during retrieval.

Regarding the effects of the duration of the maintenance period, the repeated measures ANOVA revealed a main effect of Maintenance Period Duration on TF3 factor scores [*F*(1,28) = 13.43, *p* = 0.001]. Paired comparisons showed significantly larger negative scores in the LMP condition than in the SMP condition (*p* = 0.001), which indicate significantly larger amplitude for N2 in the LMP condition than in the SMP condition (**Figure 6**).

Repeated measures ANOVA also revealed a significant interaction between Maintenance Period Duration and Memory Load on P300 latency [*F*(1,28) = 5.53, *p* = 0.026]. Paired comparisons showed significantly longer P300 latency in the SMP condition than in the LMP condition for high memory load (*p* = 0.021).

In addition, the duration of maintenance period had significant effects on brain activity between 160 and 200 ms post-stimulus during WM retrieval (*p* = 0.004). Significantly higher activation was observed in the left middle temporal gyrus (BA 39) for the LMP condition than for the SMP condition (**Figure 4C**). Coordinates of the voxels showing maximal difference, *t* values and the associated *p*, are shown in **Table 2**.

## DISCUSSION

### TASK PERFORMANCE

Task performance was modulated by memory load, but not by the duration of the maintenance period. The IE scores were longer in trials with high memory load than in trials with low memory load. In other words, task performance was poorer in the HL condition than in the LL condition, which is consistent with previous reports of decreased task performance with increased levels of memory load (Luck and Vogel, 1997; Alvarez and Cavanagh, 2004). Therefore, it seems that memory load disrupts task execution.

In the present study, maintenance period duration did not appear to affect task performance. This contrasts with previous findings of studies that used similar maintenance period durations to those applied here (i.e., 500, 2000, and 5000 ms or 1500, 3000 and 6000 ms; van der Ham et al., 2007, 2010); thus, the previous studies reported shorter RTs and lower error rates for short maintenance periods than for long maintenance periods, and the authors concluded that longer maintenance periods impair task performance. The differences in the effects of maintenance period duration may be related to the different stimuli used in these and previous studies (domino tiles vs dots) or to differences in the way that the match between the target and the probe stimulus was judged (multiple choice vs match/non-match).

### TIME COURSE OF BRAIN ELECTRICAL ACTIVITY DURING INFORMATION ENCODING AND RETRIEVAL

The main objective of the present study was to determine the ERP components that comprise the time course of electrical brain activity involved in information encoding (during sample stimulus processing) and retrieval (during probe stimulus processing) in WM. The tPCA revealed that the time course of both cognitive events (encoding and retrieval) comprises six TF, which, in turn, correspond to the following ERP components: P1, N1, P2, N2, P300, and PSW (encoding) or NSW (retrieval).

The maximal amplitude of P1 (TF6) was recorded at occipital and parietal electrodes, with a mean peak latency of 119 ms after sample stimulus onset (encoding) and 109 ms after probe stimulus onset (retrieval). This component has been associated with the detection and early analysis of low level perceptual features of the stimuli that happens in the focus of attention (Luck et al., 1990; Taylor, 2002). This low level perceptual analysis is supposed to be executed in the extrastriate visual cortex (Di Russo et al., 2001) and also to be modulated by top-down mechanisms (Clark and Hillyard, 1996).

The maximal amplitude of the N1 (TF 4) component was observed in central and parietal sites, with a mean peak latency of 130 ms after sample stimulus onset and 128 ms after probe stimulus onset. Vogel and Luck (2000) have distinguished two N1 subcomponents; one of these has a posterior distribution, which is typically observed in simple visual paradigms, and the other has more anterior distribution and is considered a correlate of preparatory processes. Given its scalp distribution, the TF 4 in the present study may be related to the anterior N1. In the previous study (Vogel and Luck, 2000), the anterior N1 was observed whenever the participants were able to carry out preparatory processes to respond to a stimulus and it was absent when the experimenters precluded such preparation (Vogel and Luck, 2000). In particular, the visual anterior N1 component may be related to an anticipatory feature set, i.e., attentional preparation to detect certain task relevant features or dimensions of the incoming stimuli (Chen et al., 2006); thus, the anterior N1 probably reflects the search for those features in the incoming stimuli. This component has also been associated with the initiation of a readjustment of this attentional setting when the task relevant features or aspects of the incoming stimuli are changed (Töllner et al., 2009).

The amplitude of the P2 (TF5) component was maximal at occipital and central electrodes, with a mean peak latency of 212 ms during sample stimulus processing, and it was maximal at central and frontal electrodes, with a mean peak latency of 205 ms, during probe stimulus processing. This component has been associated with the detection and analysis of task relevant stimulus features (Luck and Hillyard, 1994; Potts, 2004). Therefore, it has been considered as a correlate of the comparison of the incoming stimuli features with a mental template of the task relevant features, which is carried out in frontal regions, probably in the orbitofrontal cortex (Potts and Tucker, 2001; Potts, 2004).

The maximal amplitude of the N2 (TF3) component was recorded at parietal, temporo-parietal and parieto-occipital electrodes, with a mean peak latency of 264 ms after sample stimulus onset and 261 ms after probe stimulus onset. This component forms part of the N2 family (Näätänen and Picton, 1987; Folstein and van Petten, 2008), and it probably corresponds to N2c (Pritchard et al., 1991) or N2pb (Luck and Hillyard, 1994). These components have been associated with the evaluation and classification of stimuli (Folstein and van Petten, 2008).

The maximal amplitude of the P300 (TF2) component was recorded at parietal and occipital electrodes, with a mean peak latency of 381 ms after sample stimulus onset and 379 ms after probe stimulus onset. The P300 latency has been associated with the time required for stimulus evaluation (Kutas et al., 1977; Polich, 2007), whereas its amplitude has been considered a correlate of the allocation of processing resources (Kok, 1997, 2001; Polich, 2003). However, despite the large body of research on its characteristics, there is some debate about its functional role. The most widely accepted hypothesis is probably the context updating hypothesis, which suggests that P300 is a correlate of the cognitive processes that update WM stimulus context (Donchin, 1981; Donchin and Coles, 1988).

Finally, TF1 was associated with different ERP components. Thus, during sample stimulus processing it was associated with a PSW with central scalp distribution. This type of wave has been related in different studies to decision making and post-categorization processes, which vary depending on the nature of the task that the subject is performing (García-Larrea and Cézanne-Bert, 1998; Folstein and van Petten, 2011). Conversely, during probe stimulus processing it was associated with a NSW with fronto-central scalp distribution, which is probably related to motor preparation components (i.e., readiness potential or “bereitschaftspotential”). These components are characterized as long-lasting negative waves that reach their maximal amplitude during the execution of a voluntary movement (Vaughan et al., 1968; Shibasaki and Hallett, 2006).

Therefore, the tPCA revealed that the sample stimulus that has to be memorized and the probe stimulus that causes the information retrieval both triggered similar brain electrical activity.

### EFFECTS OF MEMORY LOAD ON BRAIN ELECTRICAL ACTIVITY DURING INFORMATION ENCODING

Some ERP components proved sensitive to sample stimulus memory load. Thus, a lower amplitude for P300 and larger amplitude for PSW were observed in the HL condition than in the LL condition.

The P300 amplitude was smaller in HL trials than in LL trials. Several studies have reported an inverse relationship between P300 amplitude and memory load (Kok, 1997, 2001; Polich, 2007). Therefore, it has been suggested that high memory load produces greater demands on processing resources for the maintenance and elaboration of information in WM, which, in turn, reduced the processing resources available for the processes reflected by P300, namely the updating of the stimulus context (McEvoy et al., 1998; Morgan et al., 2008). Consequently, the lower amplitude of P300 in HL trials than in LL trials may reflect fewer processing resources available for stimulus context updating (P300) due to the higher demands of processing resources exerted by other processes such as maintenance and elaboration of information.

Conversely, the PSW amplitude was larger in the HL condition than in the LL condition, which is consistent with previous reports (García-Larrea and Cézanne-Bert, 1998). This may indicate that extra stimulus processing, which is not necessary in LL trials, is required for high memory load after stimulus categorization and encoding in memory (García-Larrea and Cézanne-Bert, 1998). This also supports the suggested interpretation for the load related changes in P300 amplitude, i.e., a reduction in the processing resources available for context updating as reflected in P300 because they are allocated to the extra processing reflected by PSW.

During encoding, memory load also modulates the estimated current density distribution. Thus, in the HL condition, lower activation was observed in the right anterior cingulate and medial frontal gyrus (BA 6 and 32) than in the low memory load condition, between 96 and 128 ms after sample stimulus onset. This probably reflects a reduction in the activity of the default mode network (DMN) due to the higher demands imposed by HL than LL trials. Both of the aforementioned regions are included in the DMN, which typically appears more active during resting than during active task execution (Tomasi et al., 2006; Raichle and Snyder, 2007). Moreover, it has been shown that activation of this DMN decreases in a memory load dependent manner during active task execution (Tomasi et al., 2006; Raichle and Snyder, 2007).

### EFFECTS OF MEMORY LOAD ON ELECTRICAL BRAIN ACTIVITY DURING INFORMATION RETRIEVAL

During retrieval in WM, memory load also modulates some parameters of several ERP components. Thus, larger N2 amplitude and smaller P300 amplitude were observed in the HL condition than in the LL condition. Furthermore, P300 latency was longer in HL trials than in LL trials for the short maintenance period.

Modulation of the N2 amplitude associated with memory load (larger N2 amplitude in HL than in LL conditions) during probe stimulus processing (retrieval) may be related to comparison processes between the domino tiles that comprise the probe stimulus. During processing of the probe stimulus, the three domino tiles must be compared with the memory template of the sample stimulus and classified according to the features that they share with this template, in order to identify the tile that is identical to the sample. The process may be reflected by N2 (Folstein and van Petten, 2008). Hence, it seems that there is a higher demand on processing resources for stimulus discrimination and classification (indicated by higher N2 amplitude) when the stimulus includes more information load (more dots and more complex configural relations).

The memory load dependent modulation of the amplitude of the P300 component during retrieval is similar to that observed during encoding and, moreover, is consistent with previous findings (Kok, 1997, 2001; Polich, 2007). Therefore, it seems that during information retrieval and comparison with new visual input, there is a reduction in the processing resources available for stimulus categorization and context updating in the HL condition relative to the LL condition. This may be due to the higher demand for processing resources exerted by other processes, e.g., stimulus classification and discrimination, which, in turn, depletes the resource available to context updating (McEvoy et al., 1998; Morgan et al., 2008).

In addition, the latency of P300 was longer in the HL condition than in the LL condition in trials with a short maintenance period. The P300 latency is considered to be a correlate of the time needed for stimulus evaluation (Kutas et al., 1977; Polich, 2007). Hence, the longer P300 latency observed in HL than in LL trials for the short maintenance period may reflect the longer time required for stimulus processing when the information load on the domino tiles is high.

In summary, during information retrieval and its comparison with new visual input, high memory load produces an increase in the demands for processing resources for the discrimination and classification of stimuli, and it reduces the resources available for stimulus context updating in WM. Furthermore, for short maintenance periods, high memory load increases the time needed for stimulus evaluation and categorization.

Several memory load dependent modulations were also observed in the estimated current density distribution during information retrieval in the DMS task. These modulations span three different time intervals, when different regions showed memory load dependent variations in activation.

The first time interval extended from 148 to 164 ms after probe stimulus onset, and the activation was lower in HL than in LL at frontal areas associated with the DMN (i.e., medial frontal gyrus and anterior cingulate), as occurred during encoding (Tomasi et al., 2006; Raichle and Snyder, 2007). The lower activation was also observed in the left superior frontal gyrus, a region associated with mental introspection processes (Goldberg et al., 2006). Similarly to DMN activity, these types of introspective processes are deactivated during active task execution (Goldberg et al., 2006).

In contrast, in the right middle frontal gyrus (BA 10), greater activation was observed, between 284 and 334 ms after probe stimulus onset, in HL trials than in LL trials. The BA 10 has been associated with coordination and integration between external and internal information (Christoff and Gabrieli, 2000; Ramnani and Owen, 2004). This process is probably necessary for the comparison between the sample stimulus memory template and the probe stimulus domino tiles. Thus, in HL conditions, this comparison may require more processing resources than in LL conditions, which may have been reflected as greater activation in this region in the present study.

Finally, between 568 and 620 ms after probe stimulus onset, activation was lower at the right superior and medial frontal gyri (BA 8 and 6) for HL than for LL trials. These Brodmann areas (i.e., 6 and 8) have been associated with movement planning and execution (Sadato et al., 1997; Chouinard and Paus, 2006). Hence, this memory load related difference in activation observed in the present study may indicate that motor response preparation is influenced by this variable. This is probably due to more complex processing of high memory load than LL stimuli.

### EFFECTS OF DURATION OF THE MAINTENANCE PERIOD ON BRAIN ELECTRICAL ACTIVITY DURING RETRIEVAL

Maintenance period duration modulated brain electrical activity during information retrieval. The N2 amplitude was larger in trials with long maintenance periods than in trials with a short maintenance period. The P300 latency was shorter in the LMP than in the SMP for high memory load trials.

The larger N2 amplitude observed in trials with a long maintenance period than in those with a short maintenance period is consistent with the results of van der Ham et al. (2010). Moreover, it may reflect an extended dedication of processing resources to stimulus discrimination and classification in the LMP than in the SMP. On the basis of time-based decay theories, this result would be interpreted as a signal of a more complex comparison process due to a weaker memory trace of the target stimulus in the long than in the short maintenance period, which may, in turn, be caused by the longer time that the information must be held in mind.

The shorter P300 latency in the LMP than in the SMP in high memory load trials might indicate that less time is needed in the former condition to complete probe stimulus evaluation. This effect seems to contradict the expectations of time-based decay theories. Accordingly, mixed results were obtained regarding the assumption of a pure time-based decay theory, since they show that the effects of maintenance period duration are complex. Therefore, future work should clarify the effects of the maintenance period duration in stimulus evaluation and classification as well as on task performance.

In addition to the effects on ERP components, the duration of the maintenance period also modulates the estimated current density distribution during probe stimulus processing. Accordingly, between 160 and 200 ms after probe stimulus onset, the activation was larger at left middle temporal gyrus for the long maintenance period than for the short maintenance period. This region has been involved in visual and haptic recognition processes in humans (Kitada et al., 2013). Thus, it is possible that more processing resources are devoted to the visual recognition of the target among the three domino tiles in the probe stimulus in long maintenance period trials than in short maintenance period trials.

Two factors, practice and fatigue, might have affected the comparison of experimental conditions between the first and the second block of the present study. Concerning practice, Kramer et al. (1986) did not find practice effects in tasks in which target and non-targets are altered from trial to trial. They concluded that these conditions impede the automation of task execution due to a lack of consistency between trials. Similarly, the results of the present study did not match those expected for practice effects. Regarding the fatigue, in a previous study by our group (Lindín et al., 2004), it was found that a rest period of 3 min between blocks was enough to prevent fatigue effects in an auditory oddball task with two blocks of 500 tones each. Therefore, in the present work a 5 min rest period was set between blocks.

In conclusion, the results of the present study revealed that information encoding and retrieval in WM produced similar brain electrical activity, which can be decomposed into six TF corresponding to the ERP components: P1, N1, P2, N2, P300, and PSW (encoding) or NSW (retrieval). Changes in memory load were found to disrupt task performance and produce variations in the allocation of processing resources, thus reducing the amount of resources available for stimulus context updating during encoding and retrieval and increasing those dedicated to post-categorization processes during encoding, and to stimuli discrimination and classification during retrieval. Furthermore, memory load changes were accompanied by changes in the activation of different frontal lobe regions during both cognitive events. Additionally, the duration of the maintenance period did not affect task performance, but modulated brain activity. In particular, in contrast to short maintenance periods, more processing resources were devoted to stimulus evaluation and classification (indexed by N2 amplitude) after long maintenance periods, probably with the additional recruitment of left temporal lobe activation. Therefore, regarding time-based decay predictions, further studies are required to clarify the apparent contradiction between task performance and brain activity results, and to elucidate the effects of this variable on WM retrieval processes.

## AUTHOR CONTRIBUTIONS

Diego Pinal, Montserrat Zurrón, and Fernando Díaz contributed to the design and planning of the present study; they also cooperated in writing the manuscript. Diego Pinal was in charge of data acquisition and analysis.

## Conflict of Interest Statement

The authors declare that the research was conducted in the absence of any commercial or financial relationships that could be construed as a potential conflict of interest.
